# Phylogenetic, comparative genomic and structural analyses of human *Streptococcus agalactiae* ST485 in China

**DOI:** 10.1186/s12864-018-5084-0

**Published:** 2018-09-27

**Authors:** Rui Wang, Liping Li, Ting Huang, Yan Huang, Weiyi Huang, Xiuying Yang, Aiying Lei, Ming Chen

**Affiliations:** 1 Guangxi Key Laboratory for Aquatic Genetic Breeding and Healthy Aquaculture, Guangxi Academy of Fishery Sciences, Nanning, Guangxi 530021 People’s Republic of China; 2 Institute of Animal Science and Technology, Guangxi University, Nanning, 530005 Guangxi China; 3 Guangxi Center for Disease Control and Prevention, Nanning, 530021 Guangxi China; 4 National Medical College of Right Rivers, Baise, 533000 Guangxi China

**Keywords:** Group B *Streptococcus*, CC103, ST485, Lac.2, CadDX, Cross infection

## Abstract

**Background:**

*Streptococcus agalactiae* (Group B *Streptococcus*, GBS) is a common bacteria species infecting both human and bovine. Previous studies have shown that the GBS isolated from human and bovine are mostly unrelated and belong to separate populations. However, recently, the bovine GBS CC103 has become the dominant epidemic strain and frequently isolated from human patients. In particular, the ST485 GBS, a member of CC103, has become the new dominant ST in China and exhibited very high pathogenicity. This phenomenon is not consistent with the established understanding about the relationship between bovine and human GBS, which needs to be re-investigated.

**Results:**

The genome-based phylogenetic analysis showed that the human and bovine GBS CC103 strains had very close genetic relationship and they were alternately distributed on the evolutionary tree. CC103 strains evolved into several branches, including the ST485, which exhibited high pathogenicity and specifically infected human. Compared to other CC103 strains, the ST485 lacked Lac.2 gene structure and acquired the CadDX gene structure in their genomes.

**Conclusions:**

Our results indicate that GBS CC103 could propagate across human and bovine, and GBS ST485 might evolve from the ST103 that could infect both human and bovine. Moreover, the recombination of Lac.2 and CadDX gene structures might play an important role in the formation of highly pathogenic ST485 in China.

**Electronic supplementary material:**

The online version of this article (10.1186/s12864-018-5084-0) contains supplementary material, which is available to authorized users.

## Background

*Streptococcus agalactiae*, also called Group B *streptococcus* (GBS), is associated with early and late onset diseases in infants. It colonizes the urogenital and gastro-intestinal tracts without causing symptoms, and leads to diseases like septicemia in non-pregnant adults [1, 2]. The multi-locus sequence typing (MLST) of GBS strains isolated from different countries showed that most human-derived strains and clinical isolates were clustered into five major clone complexes (CC) (CC1, CC10, CC17, CC19 and CC23) [3], and the majority of bovine isolates belonged to another clone complex, CC67 [4]. However, recently, in some regions, the human dominant strains (such as CC1 and CC23) began to spread in bovine, and replaced CC67 to become the most widespread CC causing cow mastitis. Moreover, the newly appeared GBS CC103 strains have become the major strains causing cow mastitis in some countries of Europe and Asia [5, 6]. Recently (2015–2017), the isolation frequency of human GBS CC103 significantly increased in China (from 1.25 to 21.74%), especially the sequence type (ST) 485 (from 1.25 to 14.13%), which has become the new dominant ST and exhibits 10–20 times higher pathogenicity than the strains outside of China [7–11]. In this study, we examined the evolution of human and bovine CC103 strains by performing phylogenetic, comparative genomic and structural analyses, and investigated the origin and causes of the highly pathogenic GBS ST485 in China.

## Methods

### GBS isolates, genome sequencing and annotation

The draft genomes of 18 isolated GBS CC103 strains were determined using Illumina HiSeq2000 sequencing platforms, and then they were assembled by the ABySS program [12]. The minimal coverage was 500-fold. Since all the current public databases do not have the full-length genome data of ST485 strains, we used primer walking to close the gaps in the draft genome of ST485 strain BSE009 (one of the above 18 CC103 strains), and the resulting PCR products were sequenced to generate the whole genome. The assembled sequences were uploaded to the RAST website for gene function annotation and metabolic pathway construction (http://rast.nmpdr.org/). In addition, the genomes of another 52 GBS strains in major CC groups, which came from different hosts, were selected for evolution analysis. The strains involved in this study are listed in Additional file 1, which includes the information about their CC and ST, the capsular serotype, host, source, associated diseases, year of isolation, geographical origin, and genome accession number. The 18 CC103 strains were obtained directly from patients, and the information about the isolation procedure was reported in our previous article [11]. All subjects provided written informed consent before their inclusion in the study.

### Phylogenetic analysis and CRISPRs analysis

OrthoMCL was used to record the orthologous protein sequences among the isolates [13]. MAFFT was used to perform the multi-sequence alignment for single copy homologous proteins [14], and the poorly aligned positions and divergent regions were removed. ProtTest was used to do the Maximum Likelihood (ML) Estimation for phylogenetic tree, and the model parameters were obtained from PhyML [15]. AIC and BIC scores were evaluated to obtain the optimal amino acid substitution model. ML method was used to construct a phylogenetic tree with 1,000 bootstrap replications using RaxML software [16]. CRISPRs finder and CRISPR recognition tool (CRT) were used to identify the CRISPR sequences (clustered regularly interspaced short palindromic repeats) [17, 18]. Each unique spacer was numbered manually, and the result was analyzed and modified according to Lier et al. [19].

### Comparison of whole genome sequences and functional genes

The genome sequences and functional coding genes of GBS strains were compared by the sequence alignment and functional gene alignment functions in RAST Server [20]. The results were confirmed by comparing with public databases (nr/nt). The alignment of GBS Lac.2 and CadDX gene structures, and the construction of phylogenetic trees were performed with MEGA7 using the ML method [21].

## Results

### Genome-based phylogenic analysis found human-bovine co-infecting GBS strains

**Fig. 1:**
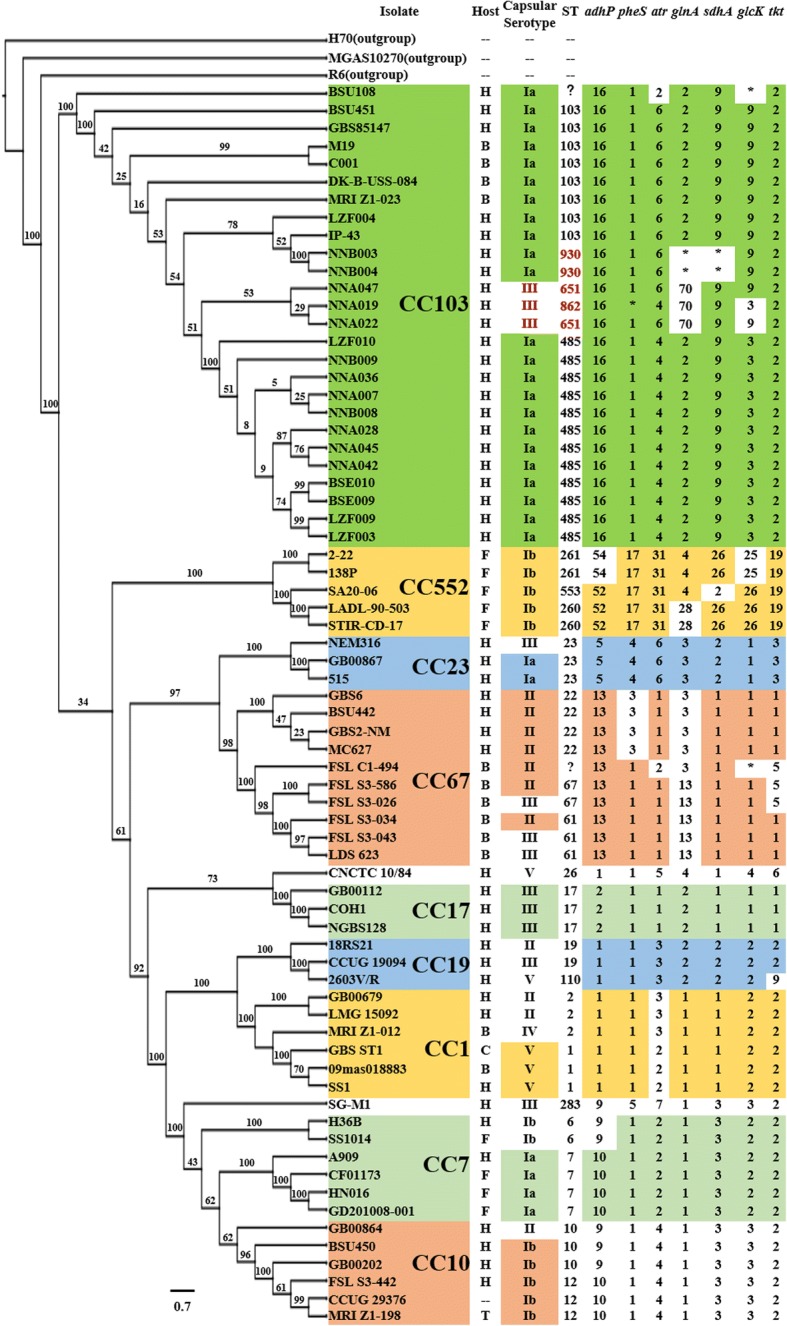
The phylogenetic relationships among GBS strains from different CC groups. On the left is the phylogenetic tree of 70 GBS strains, which was constructed with 1,000 bootstrap replications and rooted by outgroup. On the right is the information about strain host, serotype and ST. The major clonal complexes (CC) 1, 10, 17, 19, 23, 67, 102 and 552, which were defined by GBS MLST website (http://pubmlst.org/sagalactiae/), were all separated by distinct branches. H, *Homo sapiens*. B, *Bos Taurus*. F, fish. C, *Canis lupus familiaris*. T, Tursiops. *adhP*, *pheS*, *atr*, *glnA*, *sdhA*, *glcK* and *tkt* are the names of 7 allelic genes in MLST analysis, and below are the numbers of each allelic gene in MLST website. The colors (or absence of color) are used to emphasize certain regions of the chart

**Fig. 2:**
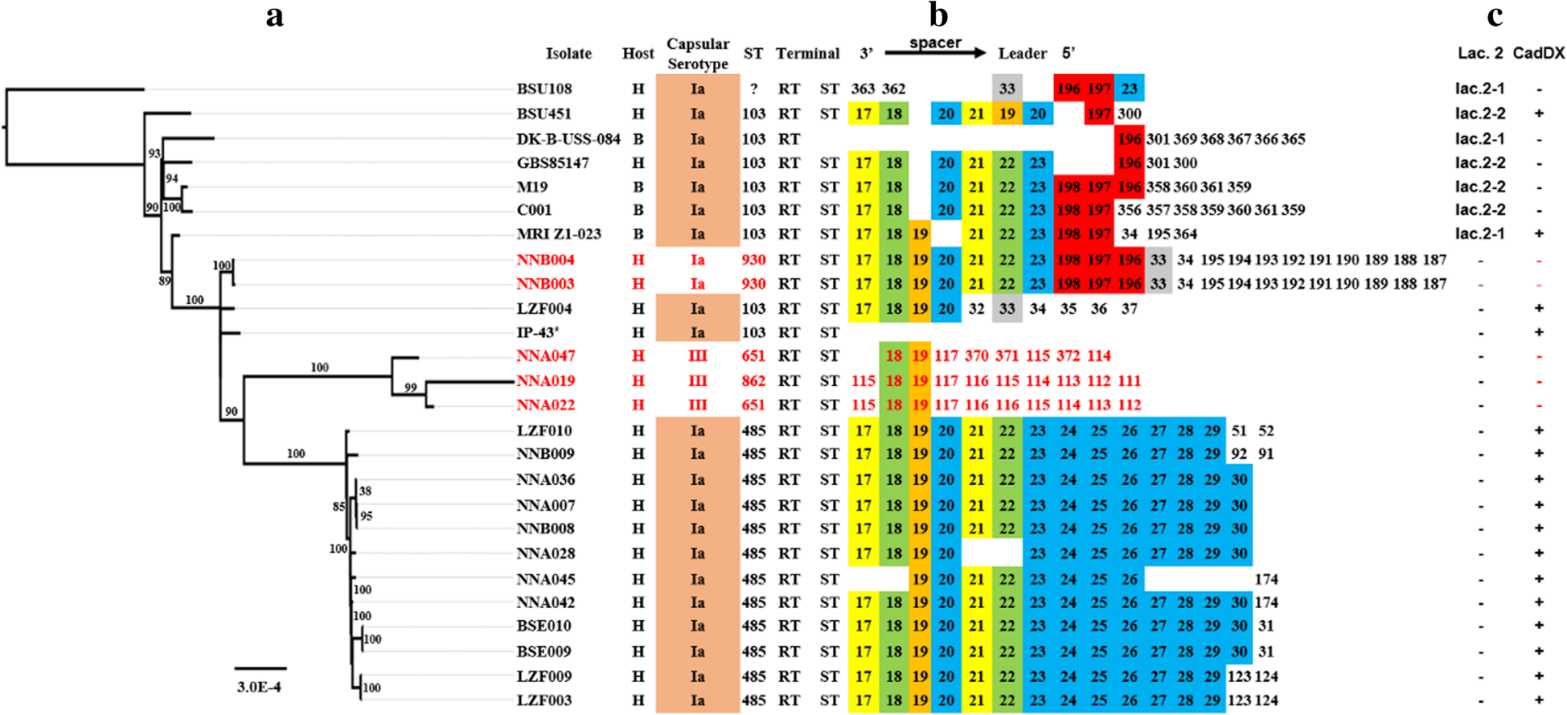
The phylogenetic analysis and gene structure comparison of 26 GBS CC103 strains. **a** The phylogenetic analysis of 26 GBS CC103 strains based on genome sequences, and the phylogeny was constructed with 1,000 bootstrap replications and rooted by the result of Fig. 1. A few strains with non-frequent ST and serotypes are highlighted in red. **b** Comparison of CRISPR1 loci. Internal repeats are not included; only terminal repeats (RT) and spacers are represented. The spacers are numbered, and same number represents the same sequence. The colors are used to emphasize the major spacers for easier viewing, and the same spacers are represented by same colors. **c** Gene structure comparisons of Lac.2 and CadDX. Lac.2–1 and Lac.2–2 are two types of Lac.2. +, the strain has this gene; −, the strain does not have this gene structure. *, there’s a gap at the CRISPR1 sequence

Considering the strain hosts and their serotypes, we chose at least 3 representative strains from each CC for phylogenetic analysis. Based on the MLST studies, a total of 70 GBS strains were selected to represent the known diversity of GBS population, which included 46 human strains, 12 bovine strains and 9 fish strains. Three strains from the genus *Streptococcus* were chosen as outgroup strains, and their genomic information was available in GenBank. The strains used in the study are listed in Additional file 1. The Maximum Likelihood (ML) method was used to construct the phylogenetic tree (Fig. 1), with the information from 96,665 amino acids of 391 single copy orthology clusters. We found that the 70 isolates were clustered into nine well-resolved lineages, which corresponded to the MLST defined CCs (Fig. 1). CC103 strains were clustered together into one branch. To more accurately reflect the evolutionary relationship within CC103 strains, we constructed the phylogenetic tree for CC103 (Fig. 2) using the information from 328,013 amino acids of 1,104 single copy orthology clusters of the 26 CC103 strains. The evolution result of CC103 strains was consistent with the CC103 ST clustering results. The bovine and human ST103 strains were alternately distributed on the evolutionary tree. Interestingly, the human dominant strain ST485 that emerged in China had a common ancestor with the bovine ST103 strain MRI-Z1–023. In addition, the ST485 strains were clustered together and had very close genetic distance to each other. Among all the isolated human CC103 clinical strains, ST485 accounted for 67% (12/18), indicating an evolutionary bottleneck for human CC103 strains.

### CRISPRs analysis further revealed the characteristics of GBS CC103 strains

CRISPRs (clustered regularly interspaced short palindromic repeats) are a bacterial adaptive immune defense mechanism to protect against the deadly outcomes from mobile genetic elements (MGEs) [22]. CRISPRs are a family of noncontiguous DNA repeats interspaced by unique spacer sequences with constant length [23]. The repeats are highly conserved within a CRISPR array, but the 3′ terminal repeat (RT) is specific for different GBS CCs. Spacer sequence corresponds to the previous exposure of MGEs, and it is inserted at the CRISPR leading end. The closer the spacer to the 3′ terminal, the more conserved the spacer sequence is [24]. Therefore, CRISPRs analysis can be used to investigate the phylogenetic relationships between subspecies [22]. There are two CRISPRs in GBS genome: Type 1-C CRISPR2, only present in a few strains, and type 2-A CRISPR1, ubiquitously present in all strains [25]. As shown in Fig. 2, except for one strain (IP43) that has sequence gap at CRISPR1, all the other 25 strains had intact CRISPR1 sequence structures. All the CC103 strains had consistent terminal repeats (RT) and terminal spacers (ST), which were also similar to the RT and ST of ST22 strains (Additional file 2). The three strains that had serotype variation exhibited the most distinct CRISPR1 sequences, with only two identical spacer sequences as other CC103 strains. There was no significant host specificity in ST103 strains, and their CRISPR sequences mostly contained No. 196–198 spacers, as compared to ST485 strains. The CRISPR1 sequences of ST485 strains were highly conserved, which again indicates an evolutionary bottleneck.

### Genomic coding sequence alignment showed mutation characteristics of GBS CC103 strains in evolution

**Fig. 3:**
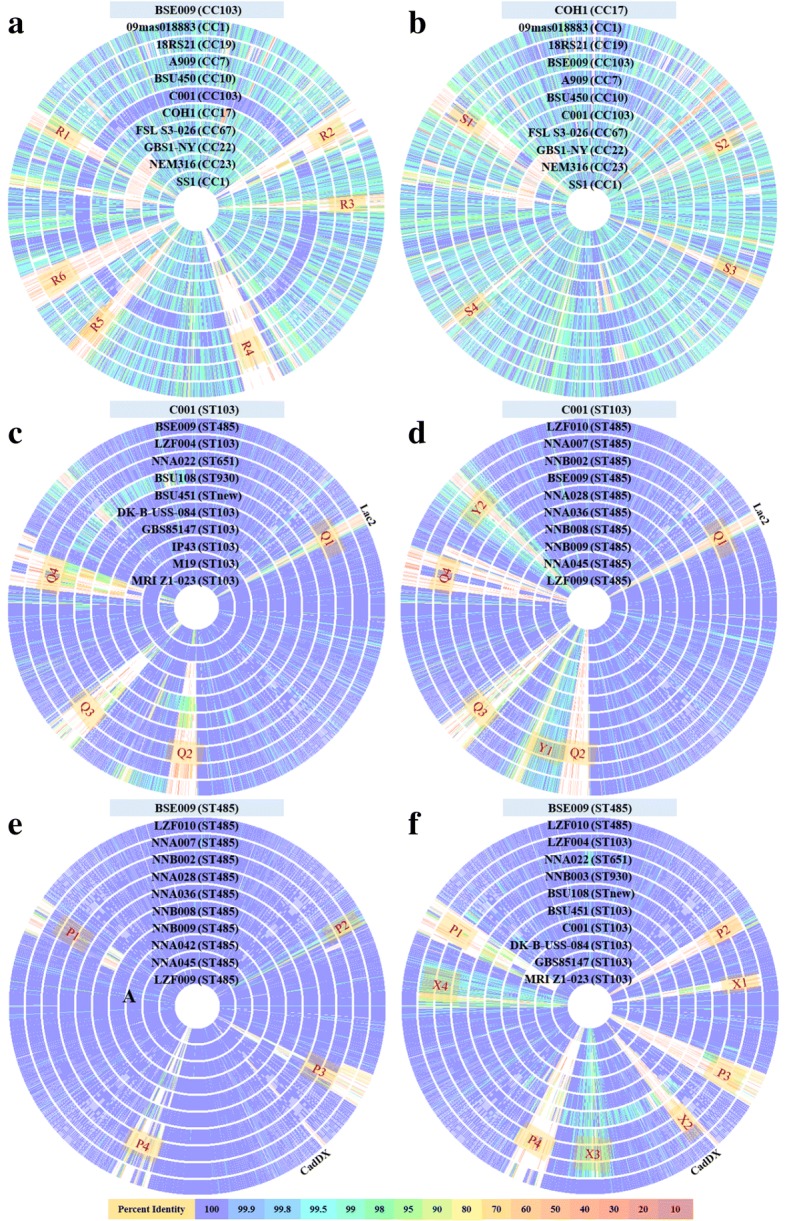
Genome coding sequence alignment of GBS strains. Each circle represents the genome of one GBS strain, and the strain name and ST/CC are labeled. The circle correspond to reference genomes are not shown. The major variation regions are numbered, such as R1, R2 ... The color bar on the bottom indicates the percentage of protein sequence identity compared to the reference genome. The positions of Lac.2 and CadDX are marked

We compared the genomic coding sequences of GBS major CCs, CC103 strains and ST485 strains, and used BSE009 (ST485), COH1 (ST17) and C001 (ST103) as the reference strains, respectively. As shown in Fig. 3, there were significant differences in gene sequences between different CCs (Fig. 3a and b). According to the genome sequence of C001 strain, there were mainly four mutant gene islands in CC103 strains (Fig. 3c). In addition to these four mutant gene islands, ST485 strains also carried two mutation enriched regions, Y1 and Y2 (Fig. 3d). Based on the genome sequence of BSE009 strain, there were four mutant gene islands in ST485 strains (Fig. 3e). In addition to these four mutant gene islands, CC103 strains also carried four mutation enriched regions (Fig. 3f). The R1-R6, S1-S4, Q2-Q4, P1-P4, X1 and X2 regions mainly contained gene deletions (Fig. 3). The sequences in these mutant gene islands were mainly coded for Phage-related proteins, hypothetical proteins with unknown function, and gene recombination related enzymes. Y1, Y2, X3 and X4 regions mainly contained point mutations and the sequence identity was generally above 97%. Most of the genes in these regions were metabolism and transport related genes. Q1 region contained both gene deletions and point mutations, and it mainly included Lac.2 (lactose operon) related genes and glucose metabolism related genes.

**Fig. 4:**
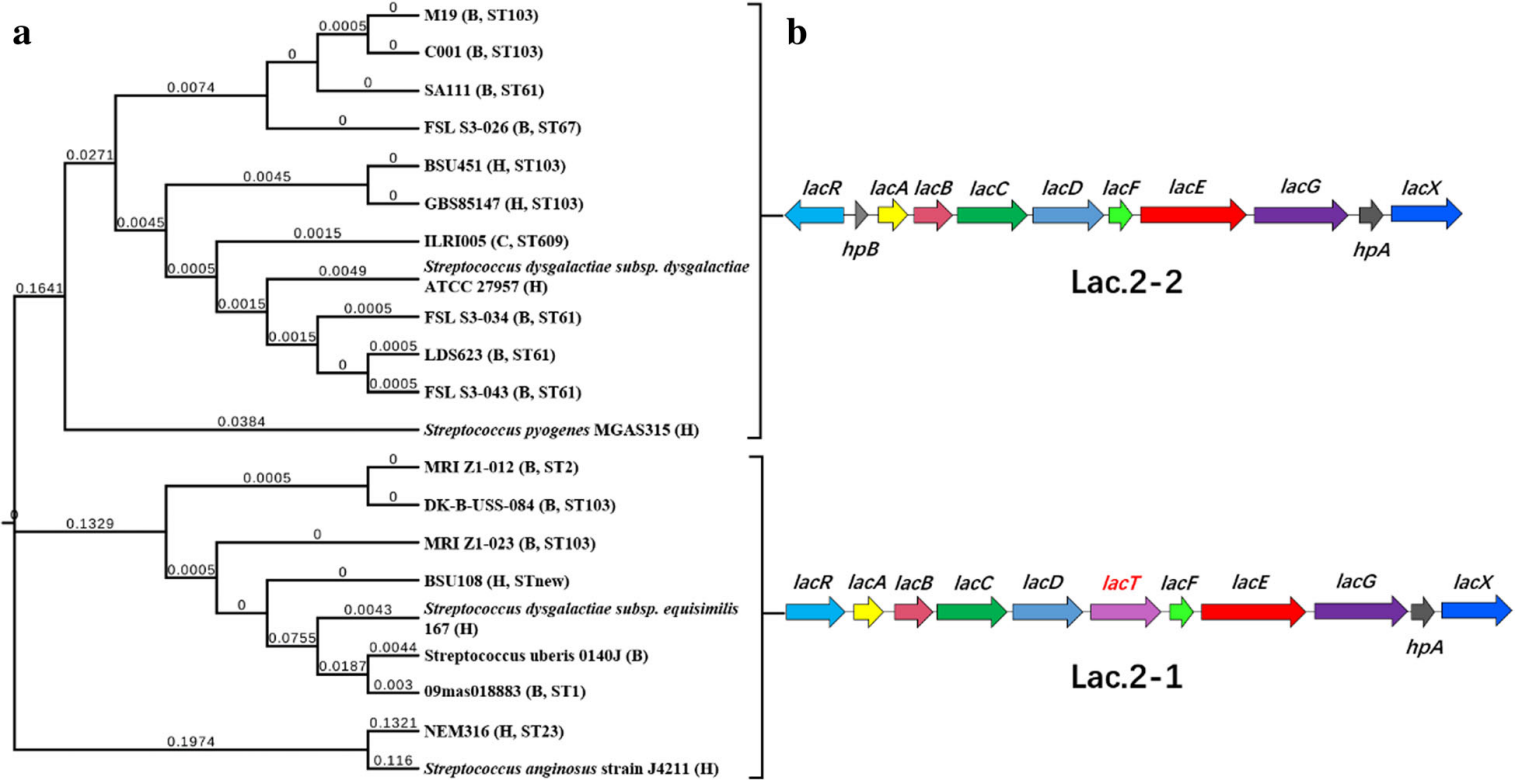
Characteristics of Lac.2 from GBS strains. **a** The phylogenetic tree of Lac.2 was constructed with MEGA7 using ML method. GBS strains are labeled with strain names, other *Streptococcus* strains are labeled with species and strain names. **b** Genetic organization of the Lac.2. The coding sequences of all genes in Lac.2–1/2 are depicted as arrows in different colors. The strains in A and B regions have Lac.2–2 and Lac.2–1, respectively. The branch lengths are labeled

### The recombination of lac.2 and CadDX gene structures may be the key factors contributing to the emergence of highly pathogenic ST485 strains

We compared the functional genes of metabolically reprogrammed CC103 strains, and screened for different gene structures. The results showed that all ST485 strains did not have the Lac.2 gene structure (a cluster of genes that are responsible for transport and lactose metabolism), but acquired the CadDX gene structure (*cadD* and *cadX* that are related to cadmium resistance) (Fig. 2). On the contrary, seven ST103 strains had Lac.2 structure and two had CadDX. We then did sequential evolution analysis on the Lac.2 gene structure of 21 GBS strains and five *Streptococcus* strains, and confirmed the sequences by comparing to public databases (nr/nt). The results showed that the Lac.2 in CC103 strains had two types, Lac.2–1 and Lac.2–2. The main difference between these two types was that Lac.2–1 had an extra *lacT* gene between *lacD* and *lacF* loci and lacked *hpB* gene between *lacR* and *lacA* loci; additionally, the directions of *lacR* genes are different between these two types (Fig. 4). As expected, Lac.2–1 and Lac.2–2 formed different evolutionary branches. From all the GBS strains we tested, only four human strains had Lac.2; all the other strains carrying Lac.2 were from bovine (11 strains) or camel (1 strain).

**Fig. 5:**
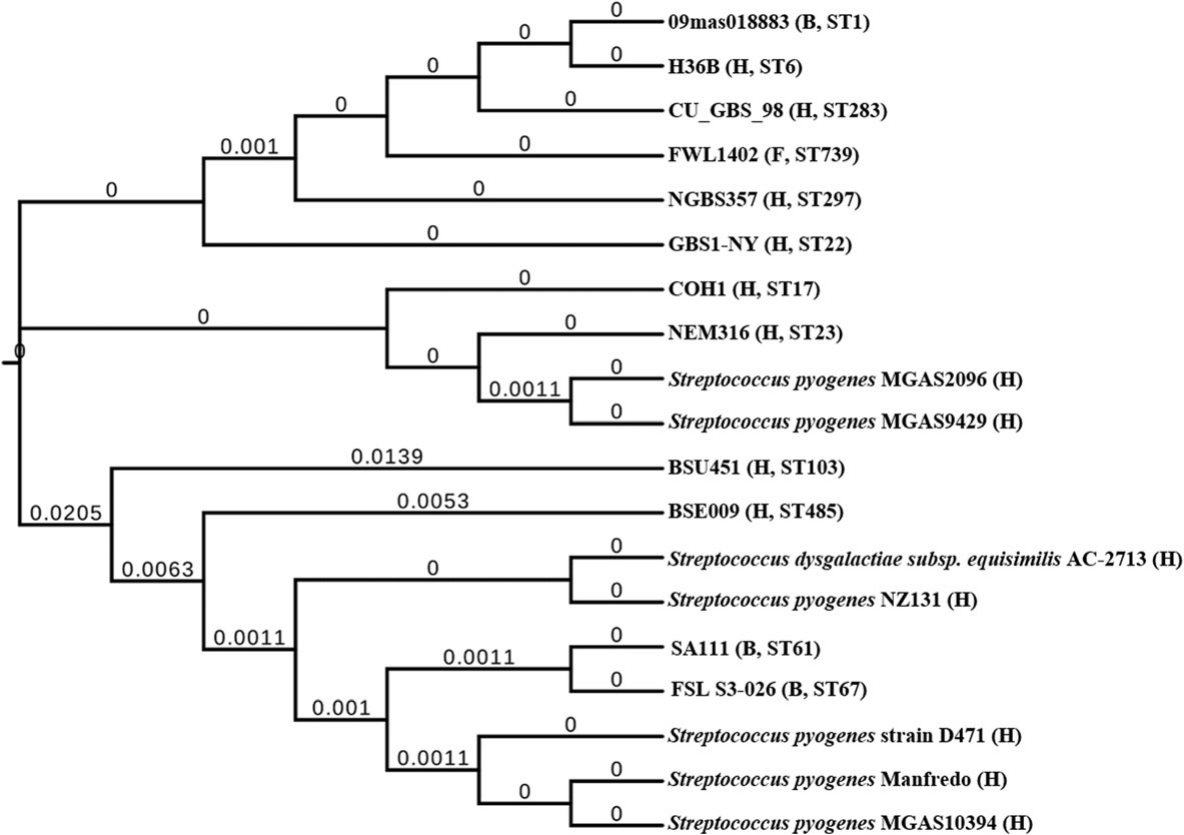
The phylogenetic tree of CadDX constructed with MEGA7 using ML method. GBS strains are labeled with strain names, other *Streptococcus* strains are labeled with species and strain names. The branch lengths are labeled

The ST103 and ST485 strains in later evolutionary branches (Fig. 2), including the MRI Z1–023, all had cadmium resistance gene structure CadDX, which encoded for cadmium resistance proteins and cadmium efflux system accessory proteins. We did phylogenetic analysis on the CadDX sequences from 12 GBS strains with different ST and seven strains from the genus *Streptococcus*. The results showed that eight GBS strains were found to be highly homologous in CadDX gene and clustered together, including ST23, ST1, ST17, ST22, ST6, etc.; but the CadDX sequences of ST485 were more similar to the bovine ST67 strains (Fig. 5).

## Discussion

GBS can infect many species, but human and bovine are the major hosts. Whether there is cross-infection of GBS between human and bovine is important for studying GBS related diseases. Previous studies showed that the GBS strains isolated from human were highly distinct from the bovine strains [4, 26–35]. However, the GBS CC103 strains were found to be able to infect many animals, including human, dog, cat, cattle, fish, etc. [5, 36–39]. Moreover, in recent years, GBS CC103 has gradually replaced the CC67 and become the dominant strain causing bovine mastitis [6]. At the same time, GBS CC103 and ST485 showed increased propagation in humans, especially in China [8, 11]. The phylogenetic analysis on GBS strains showed that GBS CC103 strains belonged to different evolutionary branches as compared to other GBS CCs, and they were clustered by themselves. Also, the human and bovine CC103 strains showed very close evolutionary relationships. These results indicate that the CC103 strains have unique evolutionary characteristics. CC103 generated multiple ST related evolutionary branches when propagated in human and bovine, including ST103, ST930, ST651, ST862 and ST485. ST103 strains can infect both humans and bovine, and they did not form independent branches in evolution, suggesting that ST103 strains did not evolve on a single host, and cross infection might happen between human and bovine. Among ST930, ST651, ST862 and ST485 strains, ST485 had the strongest infection ability [11]. We also found that ST485 could specifically infect human, which is consistent with the previous reports [8, 9, 11, 40]. Our results demonstrate a new relationship between human and bovine GBS stains, and indicate that GBS might form new, specific and highly pathogenic strain through propagating in human and bovine. However, the detailed mechanisms about how GBS propagates across human and bovine needs further investigation.

In order to study how the highly invasive ST485 appeared, we did genomic sequence alignment between GBS strains. Previous studies have found that genome recombination was a major driver for GBS genetic diversity [41, 42]. However, from the 202 invasive ST1 strains, only eight showed significant genome recombination; the remaining 194 strains only differed by 97 SNPs on average [43]. Our results also confirmed that there were a lot more mutations between different CCs, but less mutations within the same CC or ST. Compared to other CC103 strains, the genomic mutations of ST485 were enriched in several regions, and the affected genes were mainly coded for phage related proteins, hypothetical proteins with unknown function, and gene recombination related enzymes. The coding sequence alignment within the same STs showed that some of these mutations were random, and some were conserved, indicating that these mutations might affect GBS evolution, but they are not the key factors for the formation of genetic bottlenecks or the emergence and prevalence of new strains (such as ST485). For example, the key to the formation and prevalence of GBS CC17 is the tetracycline resistance gene, and the evolutionary bottleneck is the use of tetracycline, which led to a global replacement of GBS population [42]. According to the genomic coding sequence alignment and functional gene analysis, we found that Lac.2 and the cadmium resistance related genes might play a key role in the emergence and prevalence of ST485.

Carbohydrates are the most common energy source for cell growth, and thereby the carbohydrate metabolism plays an important role in the survival of prokaryotes [44, 45]. In nature, lactose is only found in mammalian milk, and is almost the only carbohydrate source in milk [46]. Therefore, Lac.2, an important gene structure for lactose metabolism, is essential for the bacteria that survive in milk. It has been shown that all the bovine GBS strains carry Lac.2 gene structure, which is necessary for lactose fermentation, while only a few human GBS strains have Lac.2 [35, 47]. The genomes of bovine and human ST1 strains have > 99% similarity, and the most significant difference was the presence of Lac.2 gene, indicating that a single genetic event can cause large phenotypic changes, such as the shift in host adaptability [43]. Our results showed that although both ST103 and ST485 strains belonged to CC103, most of the ST103 strains (both human and bovine) had Lac.2, but all ST485 strains did not have Lac.2, suggesting that the recombination of Lac.2 gene structure played a key role in GBS host adaptation.

We also noticed that the Lac.2 of GBS had two types, Lac.2–1 and Lac.2–2, and Lac.2–2 was mainly present in bovine GBS. However, the human ST103 strains GBS85147 and BSU451 had Lac.2–2, which were closely related to the Lac.2 in bovine strains. In addition, GBS85147 and BSU451 were isolated from human pharynx and respiratory tract, respectively (Additional file 1). Thus, we hypothesized that these two cases of GBS infection are possibly due to milk drinking, which leads to the propagation of bovine ST103 strains in human. Since human GBS were initially isolated from nose or throat, some studies also speculated that drinking milk might cause the infection of bovine GBS strains on human [48–50]. After pasteurized milk became commonly available, the chance of human getting infected by bovine GBS greatly reduced. However, the GBS detection rate in bulk tank milk (BTM) from dairy herds was still 90% or more [51]. Currently, there is no evidence confirming that cows are the storage pools for human epidemic GBS strains. Our results suggested that the GBS ST485 strains, which were suitable for propagation in human, might have evolved from GBS ST103, and the increasing prevalence of CC103 strains in human and bovine could be a potential threat for public health.

Whole-genome analysis of 150 bovine GBS isolates revealed that GBS CC61 had replaced the previous GBS population in Portugal, and all of the CC61 isolates acquired the mutations within an iron/manganese transporter, underlining a key adaptive strategy for colonizing in bovine host [52]. Compared to other CC103 strains, all the human ST485 strains had cadmium resistance related gene structure CadDX, which was closely related to bovine CC67 strain, but far from other human GBS strains, indicating that ST485 acquired CadDX gene structure through gene transfer. The transfer of cadmium resistance genes is often associated with the transfer of antibiotic resistance genes, which raises the possibility that antibiotic resistance and heavy-metal resistance were co-selected via the mobile genetic elements [53–55]. Cadmium pollution occurs in many countries, such as Japan, Thailand and China [56, 57]. The cadmium in soil and water are absorbed by crops, and accumulates in human body via food or drink [58, 59]. The estimated Cd in-take of each person is 30 mg per day [60, 61]. Cadmium enters bacterial cell via the transport systems that are normally used for essential divalent cations. By binding to sulfhydryl groups of essential proteins, cadmium can inhibit cell respiration and cause bacteria death [62]. With the accumulation of cadmium in environment and human bodies, GBS strains with cadmium resistance are easier to survive and spread. Therefore, we reasoned that acquiring the CadDX gene structure through horizontal gene transfer was another key factor for the emergence and propagation of ST485 strains in human.

## Conclusion

Our results suggested that: 1. GBS CC103 strains could spread across human and bovine; 2. GBS ST485 evolved from ST103; 3. The recombination of Lac.2 and CadDX gene structures played an important role in the formation of highly pathogenic GBS ST485 in China. Based on these results, the cow farm workers should be cautious about GBS infection. Moreover, further studies need to be focused on the associations between GBS strains and hosts, which will be important for the prevention and control of GBS disease.

## Additional files

Additional file 1: Sequenced strains and available genomes used in this study. (XLSX 16 kb)

Additional file 2: Inventory and distribution of CRISPR1 the terminal repeat (RT) and the terminal spacer (ST) sequences among *S. agalactiae* CC or ST. (XLSX 9 kb)

## Abbreviations

BTM: Bulk tank milk
CC: Clonal complex
CRISPRs: Clustered regularly interspaced short palindromic repeats
GBS: Group B *Streptococcus*
ML: the Maximum likelihood
MLST: Multi-locus sequence typing
ST: Sequence type
